# Effects of the therapist's statements on the patient's outcome and the therapeutic alliance: A systematic review

**DOI:** 10.1002/cpp.2416

**Published:** 2020-01-11

**Authors:** Jennifer Kadur, Jonas Lüdemann, Sylke Andreas

**Affiliations:** ^1^ Institute of Psychology University of Klagenfurt Klagenfurt am Woerthersee Austria

**Keywords:** interaction, process, psychotherapy, systematic review, therapeutic alliance

## Abstract

This systematic review summarizes articles that examined the effects of the psychotherapist's statements on the outcome of the patient and the therapeutic alliance.

The databases PsychINFO, PSYNDEX, PubMed, and PsychARTICLES were searched, and English peer‐reviewed articles were included. Participants should be adult patients with Diagnostic and Statistical Manual of Mental Disorders diagnosis who were receiving evidence‐based psychotherapy in an individual setting. Studies with a standardized, observer‐based measurement of the therapist's verbal utterances on the basis of verbatim transcripts of therapy sessions were included. Furthermore, there should be a standardized measurement of the symptom outcome or a measurement of the therapeutic alliance. The 10 included articles showed that supportive and exploratory statements and addressing aspects in the therapeutic relationship were perceived as positive regarding symptom outcome. Negative effects were particularly evident with controlling and challenging statements of the therapist. Regarding the therapeutic alliance, both positive and negative as well as nonsignificant results were obtained. The results of this review suggest that the question of which statements by therapists correlate positively or negatively with the outcome of therapy and the therapeutic alliance cannot be answered unequivocally and must be applied to more individual and specific situations.

**Key Practitioner Message**

- Supportive and exploratory statements of the therapist correlate positively with the patient's symptom outcome
- Negative correlations evident with controlling and challenging statements of the therapist
- No clear results obtained regarding effects of therapist statements on the therapeutic alliance

## 1 INTRODUCTION

Already in Rosenzweig (1936), Rosenzweig postulated that psychotherapeutic change is based not only on specific therapeutic techniques but also on overall common factors. Since then, the question *what is effective in psychotherapy?* has been frequently examined in research. Norcross (2011) investigated which factors influence the outcome of therapy. He found that the total outcome variance in psychotherapy consists of the following components: 40% cannot yet be explained, the largest explainable part (30%) is patient contribution (e.g., severity of disorder), and the second largest part, with 12%, is explained by the therapeutic relationship. Although there is little explained variance, this factor seems to play an important role in psychotherapy. According to Kim, Wampold, and Bolt (2006), an average of 8% of the outcome variance can be attributed to the therapist and 0% to the specific treatment. The importance of the therapist proved to be greater than the specific therapeutic technique. Lutz et al. (2007) found similar results regarding the importance of therapist variables. In their study, even 17% of the variance in patient improvement was explained by the therapist. The more recent meta‐analysis of Baldwin and Imel (2013) on therapist effects found that in 45 studies, 5% of outcome variance can be explained by the therapist. This may be a rather small percentage in total but large in comparison with treatment adherence (0%), for example.

### 1.1 Therapeutic alliance

Freud (1912) already wrote in his work that the relationship between therapist and patient contributes an important factor to success in psychoanalysis. Although Freud (1912) understands the therapeutic relationship as the result of the therapist's supportive techniques, in which the patient is enabled to transfer his early, unconscious relationship patterns to the therapist, Bordin (1979) later defined therapeutic alliance as the agreement of patient and therapist regarding the therapeutic goals, tasks, and the development of a therapeutic bond, a sense of unity. He speaks of a working relationship that is conceptualized across different therapy approaches. Although there are different definitions in different schools, essential elements of the therapeutic alliance as Bordin describes them remain. Because both the therapist and patient are involved in the interaction and the development of the therapeutic relationship in the therapy setting, there are accordingly various studies on the influence of both interaction partners (Hentschel, 2005).

Currently, the therapeutic relationship is one of the most frequently investigated constructs in the psychotherapeutic process (Baldwin & Imel, 2013; Flueckiger, Del Re, Wampold, & Horvath, 2018). Baldwin, Wampold, and Imel (2007) investigated the influence of patient and therapist variability in the alliance as predictors of outcome. It was shown that variability in the therapist influenced the outcome, but variability on the side of the patient did not. This means that therapists who developed stronger alliances with their patients on average achieved significantly better therapy results. Flueckiger et al.'s (2018) recent meta‐analysis also showed that the alliance–outcome relation is responsible for 8% of the variability of the treatment outcome. This result is almost exactly the same as that found in earlier meta‐analyses.

### 1.2 Therapist's statements in psychotherapy

One way to describe and take a closer look at this therapeutic relationship is to analyse the concrete interaction between therapist and patient in psychotherapy sessions and to determine what happens in the therapeutic process. Stiles (1978), for example, investigated a pattern he called “verbal response modes.” A verbal response mode is a “category of language behaviour that implies a particular interpersonal intent or microrelationship between communicator and recipient. For example, a question asks the recipient for information or support; a self‐disclosure reveals feelings, attitudes, or intentions to the recipient“(Stiles, 1978).

This concept can be interesting for psychotherapy research because it can describe the therapeutic relationship and process independent of the concrete content of communication; it is not dependent on a certain approach of therapy but is rather a common factor of psychotherapy and can provide information in a rather general way (Stiles, 1979). In addition, various therapeutic techniques and procedures in psychotherapy can also be described as verbal response modes. Different therapeutic approaches show noticeable differences in the statements used. Huber, Schmuck, and Kächele (2012), for example, investigated verbal activity in three different forms of therapy and discovered differences in therapist behaviour in line with theory, among other things that the therapist speaks significantly less in psychoanalytic therapy than in behavioural therapy. In behavioural therapy, statements and questions are used most frequently, whereas in psychoanalysis, listening accounts for a large part of communication (Huber et al., 2012). Ruiz‐Sancho, Frojan‐Parga, and Galvan‐Dominguez (2015) subjected both therapist and patient utterances to their analysis and placed them in the context of the therapeutic process. They found that regardless of the therapist, patient, or problem of the patient, there are verbal patterns that take place in clinically relevant situations. These studies show the ongoing relevance of what happens in psychotherapy.

There are already some reviews on the influence of certain therapeutic techniques and their effect on the therapeutic alliance or the therapeutic outcome (Ackerman & Hilsenroth, 2001; Hill, 1990; Hilsenroth & Cromer, 2007; Kilmann, Scovern, & Moreault, 1979). However, of these, only Hill's (1990) article deals directly with the effect of certain statements on the outcome. In addition, because studies show that the therapeutic alliance plays an important role in the therapy outcome and a previous symptom improvement can be a reason for good alliance values (Crits‐Christoph, Gibbons, Hamilton, Ring‐Kurtz, & Gallop, 2011), both aspects, the therapeutic alliance and symptom changes in the patient, should be considered.

The primary aim of the current review is to focus on research regarding the effects of certain therapist statements on the therapeutic outcome and the alliance. The concrete research questions are as follows: (a) Is there a correlation between certain statements of the therapist and the therapeutic alliance or the outcome of the patient? and (b) as a subquestion, what instruments are frequently used for examining the therapist's statements on a verbal level?

## 2 METHOD

### 2.1 Procedure

For this systematic review, the Preferred Reporting Items for Systematic Reviews and Meta‐Analyses statement (Liberati et al., 2009) was used as a methodological orientation, although this is not a meta‐analysis.

The literature search was conducted via OVID using the following databases researchers at the University of Klagenfurt have access to: PsychINFO, PSYNDEX, PubMed, and PsychARTICLES. The search was carried out using the following keywords:

1. therapist activity
2. psychotherap* AND utterance
3. psychotherap* AND verbal respon*
4. psychotherap* interaction*
5. therap* verbal acti*
6. therap* utterance
7. therap* AND utterance
8. psychotherap* AND verbal interaction
9. therapist*
10. 1 to 8 linked with “OR“
11. AND 9
12. deduplicated

Two raters (J. L. and J. K.) independently screened the titles and abstracts of the 982 hits and were able to exclude 844. Of the remaining 134 articles, the full texts were screened, and 99 articles were excluded. Reasons for exclusion are mentioned below. Ten articles for full text screening were not available; therefore, the authors were contacted. However, responses were provided by only two, and their articles did not meet the inclusion criteria. Of the included articles, reference lists were searched for other possible records by one of the two raters. A total of 102 articles were found and screened again. At the end of the screening process, 10 studies were included in the qualitative synthesis. Agreement between the two raters was good (*κ* = .81), and discrepancies were resolved by consensus.

### 2.2 Inclusion and exclusion criteria

Inclusion and exclusion criteria of this review were defined on the basis of the PICOS approach (Higgins & Green, 2008). Because the literature search took place in April 2018, there are no more recent studies in the selection. Otherwise, there were no time limits. We focused on English articles that were published in peer‐reviewed journals.

#### 2.2.1 Participants

Participants in the studies should be adult patients with a diagnosis according to the Diagnostic and Statistical Manual of Mental Disorders (American Psychiatric Association, 2000).

#### 2.2.2 Interventions

Participants were receiving evidence‐based psychotherapy in an individual setting because we were interested in the interaction in the therapist–patient dyad.

#### 2.2.3 Control group

A control group was not relevant.

#### 2.2.4 Outcome

With regard to outcome, two aspects were defined: there should be a standardized, observer‐based measurement of the therapist's verbal utterances on the basis of verbatim transcripts of therapy sessions. This restriction to session transcripts is based on the fact that we want to focus especially on verbal interaction processes without consideration of nonverbal components that can be well observed in session transcripts. As a second outcome criterion, there should be a structured measurement of the symptom outcome, which is defined as a measured effect of a therapeutic input on the patient's symptoms or a measurement of the therapeutic alliance.

#### 2.2.5 Study design

With regard to study design, the focus was on the investigation of patient–therapist dyads. Single case studies were also included as they often provide a very detailed view on the psychotherapeutic process and statements of participants.

Therefore, we excluded settings such as group therapy, family therapy, or couple therapy. Studies were also excluded that investigated only initial interviews and study analogues.

**Figure 1:**
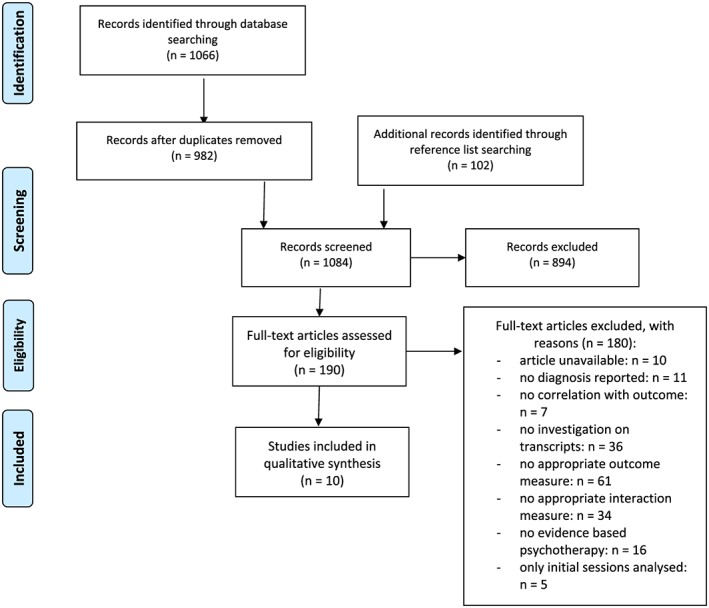
Flow diagram of the literature search

Thus the main reasons for exclusion in the full text screening were missing measurement of outcome data, analysis only of video or audio recordings of sessions rather than of transcripts, no appropriate measurement of the therapist's statements, and no analysis of evidence‐based psychotherapies. If only initial interviews were analysed, this also counted as a reason for exclusion (see Figure 1).

### 2.3 Data extraction and quality assessment

The data extraction was performed by two raters (P. H. and J. K.) independently in a structured manner. The two raters extracted data from the included studies on (a) authors, (b) title, (c) year of publication, (d) country, (e) therapy approach, (f) sample size (patients and therapists), (g) diagnoses of patients, (h) therapy setting, (i) measuring instrument of the interaction, (j) survey instrument of outcome or alliance, and (k) results. These criteria were established a priori. The results of the raters were crosschecked and in cases of disagreement, consensus was reached through discussion.

In addition, quality assessment was carried out for the included studies. The *Checklist for Assessing the Quality of Quantitative Studies* by Kmet, Lee, and Cook (2004) was used for this purpose. This tool was chosen because it assesses the quality of observational studies and nonrandomized controlled trials. It consists of 14 items, of which Items 5–7 were not used because they refer to intervention studies, which is not consistent with the included studies of this review (see Table 1). The quality of studies is assessed on the basis of a 3‐point scoring system (*not applicable*, *no* = 0, *partial* = 1, and *yes* = 2). In total, a study can therefore achieve a maximum of 22 points. An average score between 0 and 1 was calculated for each study (see Table 1).

**Table 1:** Criteria of the *Checklist for Assessing the Quality of Quantitative Studies* by Kmet et al. (2004)

Criteria	
1.	Question/objective sufficiently described?
2.	Study design evident and appropriate?
3.	Method of subject/comparison group selection *or* source of information/input variables described and appropriate?
4.	Subject (and comparison group, if applicable) characteristics sufficiently described?
5.	If interventional and random allocation was possible, was it described?
6.	If interventional and blinding of investigators was possible, was it reported?
7.	If interventional and blinding of subjects was possible, was it reported?
8.	Outcome and (if applicable) exposure measure(s) well defined and robust to measurement/misclassification bias? Means of assessment reported?
9.	Sample size appropriate?
10.	Analytic methods described/justified and appropriate?
11.	Some estimate of variance is reported for the main results?
12.	Controlled for confounding?
13.	Results reported in sufficient detail?
14.	Conclusions supported by the results?

The two raters carried out the rating independently of each other. Rater 1 (J. K.) assessed all studies, and Rater 2 (S. A.) assessed 20% of the studies to determine the reliability between the two. The interrater agreement was 100%, and each item was judged the same. The results of the quality assessment reveal that 8 of 10 studies achieved a score higher than 0.8, which demonstrates high quality of studies. The other two studies also achieved adequate to good scores (see Table 2).

**Table 2:** Characteristics of included studies in this review

Study	Patients	Diagnosis	Therapists	Therapy approach	Instruments verbal activity	Outcome/alliance measures	Results	Quality assessment score
Cunha et al., 2012	Six patients	Depression	Five therapists	Emotion‐focused therapy (EFT)	Helping skills system (HSS; Hill, 2009)	BDI	Positive effects: exploration skills (approval/reassurance, closed and open questions, restatement and reflection of feelings). Negative effects: insight skills (challenge, interpretation, self‐disclosure, and immediacy)	1
Dahl et al., 2017	Two patients	Personality disorder	One therapist	Dynamic psychotherapy	Structural analysis of social behavior‐work (SASB‐work; Benjamin, 1996)	SSCID‐II; SCL‐90	Positive effects: protecting utterances. Negative effects: therapist exercises a lot of control	0.82
Golden & Robbins, 1990	Two patients	Panic and adjustment disorder	One therapist	Time‐limited psychodynamic therapy	Vanderbilt Psychotherapy Process Scale (VPPS; Strupp, Hartley, & Blackwood, 1974)	WAI	No significant differences: high therapist exploration in low‐ and high‐alliance phases	0.68
Hayes & Strauss, 1998	30 patients	Depression	Four therapists	Cognitive therapy (CT)	Rating Scale of Therapy Change Process (TCP; Hayes et al., 1996)	Average score of BDI and HRSD; GAS	Positive effects: support and stabilizing strategies and focus on the historical antecedents of current problems	1
Hayes et al., 1996	30 patients	Depression	Four therapists	Cognitive therapy (CT)	Coding system of therapeutic focus (CSFT; Goldfried, Newman, & Hayes, 1989)	Average score of BDI and HRSD; GAS	Positive effects: focus of the therapist on direct interpersonal change and exploration of frequent patients' experiences with their parents. Negative effects: cognitive changes in the interpersonal context and positive effects: open questions and paraphrasing	1
Hill et al., 1988	Eight patients	Dysthymic, generalized‐anxiety disorder and cyclothymic	Eight therapists	Psychoanalytic treatment	Hill counselor verbal reponse modes category system (Hill, 1986); Therapist intentions list (Hill & O'Grady, 1985)	SCL‐90‐R		0.95
Jones et al., 1988	40 patients	Posttraumatic stress disorder or adjustment disorder	21 therapists	Brief dynamic psychotherapy	Psychotherapy Process Q‐Sort	BSI; BPRS	Positive effects: directive, supportive, partly psychoeducative elements, and focus on the therapeutic relationship in connection with other relationships	0.95
Lichtenberg et al., 1998	Seven patients	Depression or anxiety	seven therapists	Time‐limited therapy	Taxonomy of verbal response modes (Stiles, 1987)	WAI; SCL‐90‐R	No significant correlations with therapeutic alliance	0.95
Waldron et al., 2013	Two patients +11 patients	Personality disorder	Two therapists	Psychoanalysis	Dynamic Interaction Scale (DIS); Analytic Process Scales: “core analytic activities” (APS; Waldron et al, 2004)	PHI; RADIO‐Scales of the SWAP; GAF	Positive effects: clarifying, interpreting, and addressing defences present in the session and addressing intrapsychic conflicts	0.77
Watson & McMullen, 2005	24 patients	Depression	12 therapists	process experiental therapy (PET) and cognitive‐behavioural therapy (CBT)	Therapist Behavior Code‐Revised (TBC‐R; Bischoff & Tracey, 1995)	WAI	Low alliance sessions: therapist more supportive	1

Abbreviations: BDI, Beck Depression Inventory; BSI, Brief Symptom Inventory; BPRS, Brief Psychiatric Rating Scale; GAF, Global Assessment of Functioning Scales; GAS, Goal Attainment Scaling; HRSD, Hamilton Rating Scale for Depression; PHI, Personality Health Index; SCL‐90, Symptom Checklist‐90; SCL‐90‐R, Symptom Checklist‐90‐Revised; SSCID‐II, Structured Clinical Interview for DSM‐IV; SWAP, Shedler‐Westen Assessment Procedure; WAI, Working Alliance Inventory.

The results of the quality assessment were not used to exclude studies but to discover their strengths and limitations in comparison with the other studies.

## 3 RESULTS

The literature search resulted in 10 included articles (see Table 2). Approximately two thirds of the studies were conducted in the United States (*k* = 6), two in Europe, and one in Canada. A total of 121 patients and 61 therapists were examined, of which one sample was analysed in two reports. The patients examined most frequently suffered from depression (*k* = 5) or an anxiety disorder (*k* = 6). The most frequently applied therapy approach was psychodynamically oriented psychotherapy (*k* = 6). All studies retrospectively investigated how the therapist's statements are related to the outcome of the therapy or the therapeutic alliance. The samples examined varied greatly in size, from single case studies up to a sample size of *n* = 40 patients and *n* = 21 therapists.

### 3.1 Assessment of verbal interaction

Given the scope of the review to analyse the instruments used frequently, 12 different instruments can be found in the 10 studies. In some cases, multiple instruments were used in the same study. None of the instruments were used in more than one study, so the spectrum is correspondingly broad. The instruments are listed in Table 1.

Five of these instruments encoded the therapist's statements in nominal, mutually exclusive categories, for example, open question, approval, or information (Cunha et al., 2012; Hill et al., 1988; Lichtenberg et al., 1998; Watson & McMullen, 2005). Another six instruments captured the therapeutic statements at a slightly higher level. They summarized either concrete statements under an overarching term or aspects such as the intention of the therapist or the topic he addresses in categories (Dahl et al., 2017; Golden & Robbins, 1990; Hayes, Castonguay, & Goldfried, 1996; Hayes & Strauss, 1998; Waldron, Gazzillo, Genova, & Lingiardi, 2013). In addition, one study used the psychotherapy Process Q‐Sort, which specifies 100 items to describe the therapeutic interaction, for example, “therapist gives explicit advice and guidance” or “therapist remarks upon specific features of the interaction” (Jones, Cumming, & Horowitz, 1988).

### 3.2 Therapist's utterances and therapy outcome

Through the research question, the results can be divided into two areas with regard to outcome criteria: studies that focus on the association of the therapist's statements with the symptom outcome and studies that measure the therapeutic alliance.

There were eight studies relating to symptom outcome, for example, using the Beck Depression Inventory (Beck, Ward, Mendelson, Mock, & Erbaugh, 1961), the Symptom Checklist‐90‐R (Derogatis, 1994), or the Brief Symptom Inventory (Cunha et al., 2012; Dahl et al., 2017; Derogatis & Melisaratos, 1983; Hayes et al., 1996; Hayes & Strauss, 1998; Hill et al., 1988; Hoyt, Strong, Corcoran, & Robbins, 1993; Jones et al., 1988; Waldron et al., 2013). Two articles examined the therapeutic alliance according to the Working Alliance Inventory (Horvath & Greenberg, 1989; `Golden & Robbins, 1990; Watson & McMullen, 2005). One study covered both target criteria (Lichtenberg et al., 1998; Watson & McMullen, 2005).

Considering the overall associations of the therapist's statements on the symptom outcome of the patient, the empirical results of the studies report both positive and negative correlations (Table 3).

**Table 3:** Overview of positive and negative associations of therapist's statements with symptom outcome of the patient, sorted by study

Positive correlation with symptom outcome	Negative correlation with symptom outcome
Approval/reassurance, restatements, questions, and reflection of feelings	Challenge, interpretation, self‐disclosure, and immediacy
Open questions and paraphrasing	Therapist exercises a lot of control
Clarifying, interpreting, and addressing defences present in the session and addressing intrapsychic conflicts	Cognitive changes in the interpersonal context
Protecting utterances	
Focus on the historical antecedents of current problems	
Support and stabilizing strategies	
Focus of the therapist on direct interpersonal change	
Exploration of frequent patients' experiences with their parents	
Directive, supportive, and partly psychoeducative elements	
Focus on the therapeutic relationship in connection with other relationships	

#### 3.2.1 Positive correlations with symptom outcome

The “exploration skills,” as Cunha et al. (2012) labelled them, proved to be positive because they were applied significantly more often in good than in poor outcome cases (*r* = .78 and *r* = .83, *p* < .05). These skills include statements such as approval/reassurance, restatements, questions in an open or closed manner, and reflection of feelings. Hill et al. (1988) also found in their study of eight patients that open questions (*r* = .65, *p* < .10) as well as paraphrasing (*r* = .64, *p* < .10) are significantly related to symptom reduction. Waldron et al. (2013) could show that in patients with good outcomes, significantly more frequent core analytic activities (clarifying, interpreting, addressing defences present in the session, and addressing intrapsychic conflicts) were applied by the therapist. In addition, these therapists were more warm hearted, offered more support, and promoted more self‐esteem issues of the patient. In the two single case analyses by Dahl et al. (2017), the better outcome case also showed that the therapist and patient worked in a friendly complementarity and that the therapist helped the patient achieve greater autonomy at the end of the therapy through more protecting utterances than affirming ones. Hayes and Strauss (1998), on the other hand, concluded that the therapist's focus on the historical antecedents of current problems of the patient significantly correlates with improvement of depressive symptoms (*r* = .44, *p* < .01). They also found that a temporary destabilization of the patient ultimately led to a significant improvement in the symptoms (*r* = .46, *p* < .01). Therefore, the therapist's support and stabilizing strategies are related to a reduction of the patient's self‐protective attitude and to a positive therapeutic outcome. In another study of the same sample, Hayes et al. (1996) concluded that a focus of the therapist on the direct interpersonal change of the patient correlated with better global functioning at the end of therapy (*r* = .42, *p* < .05). Furthermore, the frequent exploration of patients' experiences with their parents was associated with improvement in depressive symptoms (*r* = .40, *p* < .05) and with better global functioning even 2 years after therapy (*r* = .44, *p* < .05).

In the study by Jones et al. (1988), various therapist statements were found to have a significant effect on the success of the therapy. The results were differentiated by whether the patients had a high or low disturbance level at the beginning of therapy. Overall, some directive (*r* = .174, *p* < .001), supportive (*r* = .138, *p* < .01), or partly psychoeducative elements (*r* = .086, *p* < .05; and *r* = .139, *p* < .01) and a focus on the therapeutic relationship in connection with other relationships (*r* = .101, *p* < .01) or on the patient's nonverbal behaviour correlated significantly with symptom reduction.

#### 3.2.2 Negative associations with symptom outcome

In addition to the positive correlations of therapist statements, statements could be found that were related with the worsening of the symptoms of patients in some studies. Cunha et al. (2012) stated in their study that insight skills (challenge, interpretation, self‐disclosure, and immediacy) frequently occurred in cases that had a poor therapeutic outcome (*r* = .16 to *r* = .19, *p* < .05). If the therapist exercises much control in the therapeutic process, especially at the end of the therapy, this can also be associated with a worse therapy result (Dahl et al., 2017). The authors explain this by the fact that the autonomy of the patient is kept low by increased control of the therapist. Lichtenberg et al. (1998) also observed that patients with poorer therapy outcomes controlled the therapy process more strongly than the therapists compared with the ratio in more positive cases. In both conditions, clients maintained significantly more control than the therapists (in good outcome cases, *F* = 5.15, *p* < .05; and in poor outcome cases, *F* = 50.37, *p* < .001). In addition, cognitive changes in the interpersonal context (*r* = −.38, *p* < .05) were negatively correlated with symptom outcome, whereas many open questions were not associated with any symptom change in the patient. (Hayes et al., 1996; Hill et al., 1988).

### 3.3 Therapist's utterances and the therapeutic alliance

The second target criterion was the correlation of therapists' statements on the therapeutic alliance. We identified three studies that addressed this question (Golden & Robbins, 1990; Lichtenberg et al., 1998; Watson & McMullen, 2005;).

In their study, Watson and McMullen (2005) found that cognitive behavioural therapists in high alliance sessions asked significantly more directive questions than process experiential therapists. The latter were more supportive. Nevertheless, for both forms of therapy, they intervened in low alliance sessions significantly more supportively than in high alliance sessions (*F* = 4.36, *p* < .05). Golden and Robbins (1990) examined the course of the assessed therapeutic alliance and the frequency of certain therapist statements in the initial, middle, and final phases of the therapies on the basis of two individual cases. In both cases, they found very high therapist exploration across all three phases, resulting in a high–low–high pattern of alliance in both patients. In addition, lower patient participation was observed in the first two phases.

Lichtenberg et al. (1998) could not find any significant correlations between the statements of the therapists and the therapeutic alliance.

## 4 DISCUSSION

The purpose of this systematic review was to examine the effect of the therapist's statements on the outcome of therapy and the therapeutic alliance, as well as the instruments used to assess these statements. In general, it should be noted that sample sizes vary widely, from individual case studies (Dahl et al., 2017; Golden & Robbins, 1990) to studies considering 21 therapists (Jones et al., 1988).

### 4.1 Issues regarding assessment instruments

The ten studies included used a total of twelve different tools to assess the therapeutic actions. This shows a wide range of possible survey instruments, which were also applied in the studies included here. Gumz, Treese, Marx, Strauss, and Wendt (2015) also found a variety of different instruments in their systematic review, in which they looked for available instruments for assessing verbal psychotherapeutic technique. These were very heterogeneous in their theoretical orientations and the definitions of verbal activity assessed, as well as in the level at which the interventions were described (global descriptions or microanalytic level). The same is given in this review: the instruments used vary in the level at which they assess therapeutic statements and the theoretical orientation to which they are subject. This should, of course, be kept in mind when interpreting the results.

### 4.2 Issues regarding the correlation of therapeutic statements with symptom outcome

Concerning the correlation of therapist statements with the therapy outcome, the results could be divided into those that showed positive and negative associations, although the results are not entirely clear. Overall, statements that had a supportive and stabilizing impact, exploratory procedures, and statements that addressed aspects in the therapeutic relationship were associated positively with symptom reduction (Cunha et al., 2012; Dahl et al., 2017; Hayes et al., 1996; Hayes & Strauss, 1998; Hill et al., 1988; Jones et al., 1988; Waldron et al., 2013). Hayes and Strauss (1998) argue that if the patient is given a safe and supportive setting by the therapist, he can better make changes and override self‐protection mechanisms. Weiss, Sampson, and The Mount Zion Psychotherapy Research Group (1986), for example, also assume in their theory, the control‐mastery theory, that a patient must first feel safe in psychotherapy to be able to make progress and open up.

Negative correlations with the success of the therapy were particularly evident with rather controlling and challenging statements of the therapist. However, the classification of interpretations is unclear. These findings show both positive and negative associations with the symptom burden of the patients. It should be noted that different patient groups with different diagnoses were investigated in the considered studies, mainly patients with depression or anxiety disorders. Therefore, these effects may also indicate that different therapeutic actions may be effective for different diagnoses. This effect in different diagnoses should be considered in future research.

Cunha et al. (2012) also observe that the applied therapeutic approach plays a role in the interpretation of the results. For example, psychodynamic psychotherapists speak significantly less than do behavioural psychotherapists (Huber et al., 2012). This also influences the type of verbal statements as well as their significance and effects. Not only the extent of verbal activity differs in different therapeutic approaches but also the therapeutic techniques. As a result, different statements are used, as Huber et al. (2012) demonstrate, and conclusions about general correlations must be drawn carefully in this review and need further research.

Overall, however, exploration skills, as summarized by Cunha et al. (2012), including approval, exploration, and reflections, proved to be related with positive therapeutic outcome as studies in this review show.

### 4.3 Issues regarding the effect of therapeutic statements on the therapeutic alliance

The connections between therapeutic statements and the therapeutic alliance are not as obvious. It turns out that supportive interventions were found at high alliance levels and that much exploration also contributes to high but also to low alliance scores (Golden & Robbins, 1990; Watson & McMullen, 2005). However, no significant results were achieved, which does not indicate a strong correlation (Lichtenberg et al., 1998).

Good alliance values in supportive and reinforcing statements on the part of the therapist do not seem surprising, as less relationship‐threatening interventions take place, and the therapeutic relationship is experienced as comfortable. Jones et al. (1988) also argue that therapeutic interventions can strongly define the relationship. A therapist who often offers encouragement and support creates a very different kind of therapeutic relationship than a therapist who, for example, is strongly focused on the patient's affects. In this respect, therapeutic interventions are closely linked to relationship factors, such as the therapeutic alliance.

### 4.4 Limitations and outlook

This systematic review also has some limitations. It is possible that not all articles on the topic were recorded with the keywords used. In addition, unpublished manuscripts were not included, and only publications in English were included. Of course, the focus on studies that collect the statements only on the basis of transcripts and by observer‐based instruments also limits the number of results. On the other hand, an investigation on the basis of transcripts seems to us to be objective and appropriate for the construct raised, the verbal utterances of therapists. Another point of weakness could be the not very narrow definition of verbal utterances. This was done deliberately in order not to limit the range of results too much, as the above criteria already considerably limit the results. As a result, it must also be accepted that the survey instruments of verbal statements are, to some extent, very different. However, it was important for this review to determine whether the effects of these statements on the therapy outcome and the therapeutic alliance could be demonstrated.

The results of this review suggest that the question of which statements by therapists have a positive or negative correlation with the outcome of therapy and the therapeutic alliance cannot be answered unequivocally. Some of the authors dealing with this issue also pointed out that using an immediate outcome measure rather than the symptom outcome as a target criterion might produce more specific results (Hill et al., 1988). Furthermore, the timing of therapeutic statements plays a role because not every intervention is helpful at every point in time. Nor does the frequency of interventions count but their quality. In general, individual differences between patients must always be taken into account (Hill et al., 1988). These are aspects that should be taken into account in future research.

## FUNDING INFORMATION

This research did not receive any specific grant from funding agencies in the public, commercial, or not‐for‐profit sectors.

## CONFLICT OF INTEREST

No potential conflict of interest was reported by the authors.
